# Evaluation of Selected Parameters of Oxidative Stress and Adipokine Levels in Hospitalized Older Patients with Diverse Nutritional Status

**DOI:** 10.3390/antiox12030569

**Published:** 2023-02-24

**Authors:** Katarzyna Mądra-Gackowska, Karolina Szewczyk-Golec, Marcin Gackowski, Alina Woźniak, Kornelia Kędziora-Kornatowska

**Affiliations:** 1 Department of Geriatrics, Faculty of Health Sciences, L. Rydygier Collegium Medicum in Bydgoszcz, Nicolaus Copernicus University in Torun, Skłodowskiej Curie 9 Street, PL–85094 Bydgoszcz, Poland; 2 Department of Medical Biology and Biochemistry, Faculty of Medicine, L. Rydygier Collegium Medicum in Bydgoszcz, Nicolaus Copernicus University in Torun, Karłowicza 24 Street, PL–85092 Bydgoszcz, Poland; 3 Department of Toxicology and Bromatology, Faculty of Pharmacy, L. Rydygier Collegium Medicum in Bydgoszcz, Nicolaus Copernicus University in Torun, A. Jurasza 2 Street, PL–85089 Bydgoszcz, Poland

**Keywords:** adipokines, older adults, GNRI, leptin, malnutrition, malondialdehyde, MNA, oxidative stress, resistin

## Abstract

Malnutrition is classified as one of the Giant Geriatric Syndromes. It carries serious consequences, such as sarcopenia or depression, which lead to functional disability. The main objective of this study was to identify parameters of oxidative stress and adipokines, which may be potential biomarkers of malnutrition in hospitalized older patients. During the study, selected parameters were determined in 137 senile patients, taking into account their nutritional status determined according to the Mini Nutritional Assessment (MNA), as well as an additional tool, namely the Geriatric Nutritional Risk Index (GNRI). Leptin and resistin were determined as the parameters with statistically significant differences between the patients classified according to the MNA. This phenomenon was confirmed using the GNRI classification. However, additional parameters for which differences were observed include the oxidized low-density lipoprotein level and activity of glutathione peroxidase. In conclusion, the determination of the mentioned markers in hospitalized senile patients as an adjunct to the routine assessment of nutritional status might be suggested to identify the early risk of malnutrition so that a personalized nutritional therapy can be implemented as early as possible.

## 1. Introduction

Malnutrition is a serious medical concern in senile patients, affecting the length of stay in hospital, treatment costs, the number of infections, the frequency of complications, and mortality [1]. The nutritional status of older patients often deteriorates during hospitalization due to increased need for nutrients, aversion to certain foods, loss of appetite, nausea, or problems with oral food intake. Despite the high incidence of malnutrition in hospitalized older adults and the availability of recognized methods to identify malnourished patients, screening is still carried out insufficiently and malnutrition is rarely identified in healthcare facilities. The prevalence of malnutrition in hospitalized older patients oscillates between 30 and 90% in relation to the method of identification [1,2,3].

Malnutrition, as one of the Giant Geriatric Syndromes, is a condition related to the aging process, diseases, and medications taken, as well as the environmental factors. In older patients, a decrease in the rate of basic metabolism and total energy expenditure, salivary secretion disorders leading to dysphagia, difficulties in forming a food bite, and impairment of the senses of smell and taste (atrophy of taste buds) are frequently observed. Diseases contributing to malnutrition include cancer, heart failure, chronic obstructive pulmonary disease, chronic kidney disease, hyperthyroidism, depression, stroke, Parkinson’s disease, dementia, as well as other conditions in which the patient has dysphagia, nausea, vomiting, diarrhea, or constipation. Appetite, feeling of hunger and satiety, and the occurrence of nausea may be affected by pharmacotherapy, which may influence the amount of food consumed [4,5]. Excessive polypharmacy, including overprescription of proton-pump inhibitors (PPIs), may increase the risk of serious health conditions. Up to 14% of depression cases, according to Laudisio et al. [6], could be avoided by withdrawal of PPIs in senile patients. Mood disorders, especially late-life depression, are especially common among senile institutionalized patients. More than one third of nursing home residents are prescribed with antidepressants, predominantly selective serotonin reuptake inhibitors (SSRI), which may affect the appetite [7]. The environmental causes include the lack or non-use of dentures, unattractive form of serving meals, and unfavorable socio-economic conditions limiting the purchase of certain food products or self-preparation of meals [4].

Malnutrition that has not been identified and/or nutritional management that has not been implemented belong to conditions dangerous for a senior and can have serious consequences [4]. The quality of life deteriorates, treatment of seriously ill people becomes more difficult and can lead to increased mortality, muscle mass and strength, as well as psychomotor efficiency, decrease noticeably, promoting gait instability and increasing the risk of falls. The disadvantageous health changes include disturbances in the function of the digestive system, disturbed respiratory system, an increase in the risk of pneumonia, problems with the circulatory system, impaired consciousness, water-electrolyte disturbances, susceptibility to bedsores and infections due to the decreased liver synthesis of proteins, hypochromasia, and osteoporosis caused by an insufficient supply of calcium and vitamin D [8]. A number of adverse consequences of malnutrition necessitate the assessment of the patient’s nutritional status at an advanced age in order to implement an appropriate nutritional intervention.

Oxidative stress accompanies many age-related diseases, such as chronic obstructive pulmonary disease, acute and chronic kidney disease, cardiovascular disease, type 2 diabetes, neurodegenerative diseases (especially Alzheimer’s and Parkinson’s diseases), or cancer [9,10]. Moreover, it is comprehensively described in sarcopenia and the frailty syndrome. The abovementioned diseases are associated with impaired activity of antioxidant enzymes and/or excessive generation of reactive oxygen and nitrogen species (RONS). It is postulated that senile dementia is also associated with the pro- and antioxidative imbalance. Various types of oxidative stress biomarkers that can supply vital information on the role of oxidative stress in the pathogenesis of different clinical conditions as well as the aging process have been identified [11]. According to numerous observations, there is a relationship between oxidative stress, including lipid, protein, and DNA peroxidation, endogenous antioxidants and total antioxidant activity, and nutritional status, such as poor nutrition, overweight, and exogenous antioxidant intake, in older adults. Evidence shows that there is a negative correlation between oxidative stress and nutritional status in older individuals [9]. Oxidative stress, cell aging, and the senescence-associated secretory phenotype (SASP) participate in the pathogenesis of abovementioned diseases, which are also related to the inflammatory state mediated by, among others, interleukin 1α (IL-1α), interleukin 6 (IL-6), and interleukin 8 (IL-8) [11]. Free radicals have a crucial role in the immune response, and severe nutrient deficiency leads to increased oxidative stress in the intracellular space. In turn, chronic oxidative stress leads to the activation of the immune system. Thereby, a vicious circle is formed, in which continuing oxidative stress and inflammation exacerbate each other, thus increasing morbidity and age-related mortality. On the one hand, there is no clinical evidence for the effectiveness of administering antioxidants as dietary supplements; however, on the other hand, seniors are recommended to eat fruits and vegetables and are encouraged to maintain a typical body mass index (BMI) between 23 and 28 kg/m^2^ [9,12,13].

Recently, adipokines, substances produced specifically by adipocytes, whose synthesis and secretion are regulated by nutritional status, have become a target of interest in studies involving older adults [14,15,16,17]. Leptin, acting on the central nervous system, regulates food intake. Low concentration of leptin stimulates and high concentration suppresses appetite. In addition, leptin increases the secretion of cytokines dependent on lymphocytes Th1, stimulating the development of inflammation, activates monocytes, and lowers insulin levels [18,19]. Resistin induces the synthesis of pro-inflammatory cytokines, such as tumor necrosis factor α (TNF α) and IL-6, and also promotes insulin resistance, mainly in hepatocytes, by activating gluconeogenesis enzymes and enhancing glycogenolysis [20]. Adiponectin is one of the most important adipokines secreted by adipose tissue, which has an anti-inflammatory effect. Its beneficial effect on the metabolism of carbohydrates and fatty acids is observed by lowering the concentration of triacylglycerols, free fatty acids, and glucose in the blood, as well as by enhancing the action of insulin. Moreover, by inhibiting the transformation of macrophages into foam cells, adiponectin has also an anti-atherosclerotic effect [20,21]. Melatonin, a hormone secreted by the pineal gland, has a multidirectional effect, such as the regulation of circadian cycles and energy metabolism. Moreover, its role in oxidative stress is emphasized as an excellent antioxidant and a factor regulating the functioning of the immune system. It was also found that melatonin normalizes the expression and secretion of two abovementioned adipokines, namely leptin and adiponectin [21].

The aim of the present contribution was to determine the relationship between the nutritional status of hospitalized senile patients and selected oxidative stress markers and adipokine levels, and to assess whether any of those parameters could be used as biomarkers of malnutrition, so that measuring their levels or activities might facilitate early identification of malnourished patients during hospitalization.

## 2. Materials and Methods

### 2.1. Participants

The presented cross-sectional observational study included 137 older adults (96 women and 41 men), who were hospitalized at the Geriatrics Clinic of the Antoni Jurasz University Hospital No. 1 in Bydgoszcz, Poland, as part of a comprehensive geriatric assessment. The mean age of the examined patients was 80.5 years (SD ≈ 7.78). The study was approved by the Bioethics Committee of Ludwik Rydygier Collegium Medicum in Bydgoszcz, Nicolaus Copernicus University in Toruń, Poland (No. KB 134/2016). All participants were informed about the principles and purpose of the study and signed an informed consent to participate in the study. In the case of patients with moderate dementia, written consent to participate in the study was also given by relatives or guardians of the patients. The inclusion criteria comprised the age of 65 years and above and the somatic condition enabling a full examination with the use of selected scales. The exclusion criteria included inability to move independently, bedridden state, Parkinson’s disease, cancer, acute illness, as well as deep cognitive disorders preventing full contact with the patient (Mini–Mental State Examination (MMSE) score below 10).

### 2.2. Study Design

The study involved interviewing, also using questionnaires, including the full Mini Nutritional Assessment (MNA) test, and collecting blood samples for the determination of selected parameters of oxidative stress and adipokine levels. Blood samples for biochemical tests were collected by qualified medical professionals in the morning (between 8:00 a.m. and 9:00 a.m.) after overnight fasting from the cubital vein into three polypropylene tubes. Two tubes (vol. 4 mL each) with a clotting activator were used to obtain blood serum, and another tube (vol. 4 mL) containing K_2_EDTA allowed the preparation of blood plasma and erythrocytes. One out of the two collected tubes in order to obtain serum was immediately transported to the Laboratory Diagnostics Unit of the Antoni Jurasz University Hospital No. 1 in Bydgoszcz, Poland, for the measurement of albumin. Other tubes were directly transported under reduced temperature condition to the Department of Medical Biology and Biochemistry at the Collegium Medicum in Bydgoszcz of the Nicolaus Copernicus University in Toruń, Poland, and centrifuged (6000× *g* for 10 min at 4 °C). Then, the blood serum and plasma samples were separated. The serum specimens were stored at −80 °C for further analysis, and the plasma samples were directly used to determine the concentration of malondialdehyde (MDA). The blood cells remaining after centrifugation were washed three times with phosphate-buffered saline (PBS) at a ratio of 1:3 and each time centrifuged (6000× *g* for 10 min at 4 °C) to remove leukocytes and thrombocytes. The red blood cells were then mixed with the PBS solution, resulting in a suspension of erythrocytes with a 50% hematocrit index, and immediately used to measure the activity of the antioxidant enzymes and the concentration of MDA.

In order to grade the nutritional status of the participants, the MNA questionnaire was utilized as an integrated part of comprehensive geriatric assessment. The MNA test consists of simple measurements and brief questions that can be performed in less than 10 min. It is divided into four sections as follows: (1) anthropometric measurements (weight, height, and weight loss), (2) global assessment (six questions concerning lifestyle, medication, and mobility), (3) dietary questionnaire (eight questions, linked to a number of meals, food and fluid intake, and autonomy of feeding), and (4) subjective assessment (self-perception of health and nutrition) [22]. The sum of 18 questions can provide a maximum score of 30. On the basis of the MNA score, the patients involved in the study were divided into three groups: adequate nutritional status, at risk of malnutrition, and protein-calorie malnutrition (Table 1).

Additionally, the Geriatric Nutritional Risk Index (GNRI) was used as a complementary tool to the MNA questionnaire. This method is even simpler and more efficient than the MNA in assessing the nutritional status of senile patients. GNRI is calculated by using the following equation:

GNRI = [1.489 × albumin (g/L)] + [41.7 × (weight/WLo)]

where WLo means an ideal weight calculated using the following the Lorentz formula:

For male: height (cm) − 100 − [(height in cm − 150)/4]

For female: height (cm) − 100 − [(height in cm − 150)/2.5]

When the “weight/WLo” is equal to or greater than 1, the ratio is set to 1 [23]. The interpretation of the GNRI index was carried out in accordance with the classification presented in Table 2 [24].

### 2.3. Biochemical Analysis

The activity of selected antioxidant enzymes was determined in a freshly prepared erythrocytic suspension with the use of spectrophotometric methods. Activity of Zn/Cu-superoxide dismutase (SOD-1; EC 1.15.1.1) was measured according to the Misra and Fridovich method [25]. Analysis was based on the inhibition of adrenaline oxidation to adrenochrome in alkaline solution at 37 °C, which induced a change in the absorbance at 480 nm. A unit of SOD-1 activity was defined as the amount of enzyme that causes 50% inhibition of the reaction with a maximum increase in absorbance of 0.025 units per minute during a directly proportional step of adenochrome formation. Activity of SOD-1 was expressed in IU/g Hb. Catalase (EC 1.11.1.6) activity was determined by the Beers and Sizer method [26], measuring the decrease in the absorbance at 240 nm of a solution of hydrogen peroxide decomposed by the enzyme at 37 °C. CAT activity was expressed in IU/g Hb. Activity of cytosolic glutathione peroxidase (GPx; EC 1.11.1.9) was assayed using the method of Paglia and Valentine [27]. The principle of the GPx activity measurement method is based on the ability of the enzyme to reduce hydrogen peroxide while simultaneously oxidizing reduced glutathione (GSH) as a coenzyme at 37 °C. The oxidized glutathione (GSSG) produced by GPx is then reduced by glutathione reductase (GR) using reduced nicotinamide adenine dinucleotide phosphate (NADPH). The formation of NADP^+^ from NADPH during this step is accompanied by a change in absorbance measured at 340 nm. Activity of GPx was expressed in IU/g Hb. Erythrocytic and plasma MDA concentration was determined with the method of Buege and Aust [28] in the modification of Esterbauer and Cheeseman [29]. The MDA concentration was measured as the concentration of thiobarbituric acid-reactive substances (TBARS), determined at 532 nm at room temperature. The MDA concentration in erythrocytes was expressed in nmol/g Hb and in blood plasma in nmol/mL. The commercially available enzyme assay kits were used to determine the serum concentrations of adiponectin (HUMAN ADIPONECTIN ELISA, HIGH SENSITIVITY, BioVendor, Brno, Czech Republic), leptin (HUMAN LEPTIN ELISA, Clinical Range, BioVendor, Brno, Czech Republic), melatonin (ELISA Kit for Melatonin (MT), Cloud-Clone Corp., Houston, TX, USA), oxidized low-density lipoprotein (ox-LDL/MDA Adduct ELISA, Immundiagnostik, Bensheim, Germany) and resistin (HUMAN RESISTIN ELISA, BioVendor, Brno, Czech Republic). The storage time of the frozen serum samples did not exceed the period indicated by the manufacturers of the ELISA kits used, allowing for reliable results. The measurements were performed according to the manufacturer’s instructions. All reagents necessary for the study, standard concentration analytes, blanks, and controls were provided in the ELISA kits used. The principle of the assay is to bind the antigen by specific anti-human monoclonal antibodies that coat the wells of a microplate. The antigen concentration was determined using the calibration curve prepared simultaneously with the test in the analyzed samples.

### 2.4. Statistical Analysis

Statistical analysis of the results was carried out by means of the Statistica 13.3 (TIBCO Software Inc., Palo Alto, CA, USA). Statistical analysis encompassed Student’s *t*-test for independent samples and Shapiro–Wilk test to test the hypothesis of normal distribution. The requirements for a one-factor analysis of variance were not met, and, for that reason, Kruskal–Wallis test was used as a nonparametric alternative to evaluate the equality of variances for a variable calculated for two or more groups. The level of significance was set at *p* < 0.05.

## 3. Results

Among the 137 participants, the average age was 80.5 ± 7.78 years, 70.0% females and 30.0% males. As many as 47 (34.31%) people were malnourished, 12 patients (8.76%) were at risk of malnutrition, and 78 patients (56.93%) were characterized by adequate nutritional status according to the MNA score. In turn, based on the GNRI score, 12 patients (8.76%) at high risk, 17 patients (12.41%) at moderate risk, and 21 patients (15.33%) at low risk of nutrition-related complications were identified. The condition of 87 patients (63.50%) did not indicate the risk of nutrition-related complications.

### 3.1. Selected Oxidative Stress Parameters and Adipokines According to the MNA

In the case of the nutrition status assessment using the MNA score (malnutrition, at risk of malnutrition, adequate nutritional status), the parameters for which statistically significant differences were determined include leptin and resistin. These variables are marked in yellow in Table 3. The other tested parameters did not show any differences with the assumed significance level.

In order to determine precisely between which groups the differences were found, a comparison of medians using box–whisker plots was performed in the further part of the analysis.

**Figure 1:**
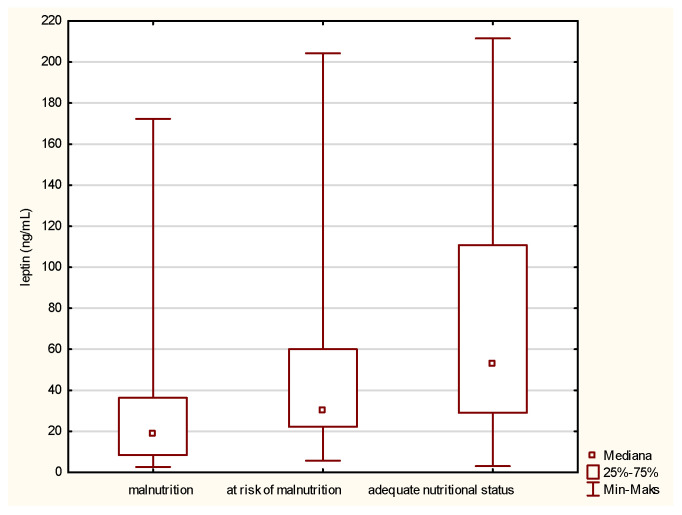
Concentration of leptin in the older patients with different nutritional status identified by the Mini Nutritional Assessment (MNA).

Leptin is the first parameter that differs between malnourished patients and those having a normal nutritional status. The comparison of the medians, presented in Figure 1, revealed a statistically significant increase in the leptin concentration in the case of people with a normal nutritional status, which means a decrease in its level along with a deepening of malnutrition.

**Figure 2:**
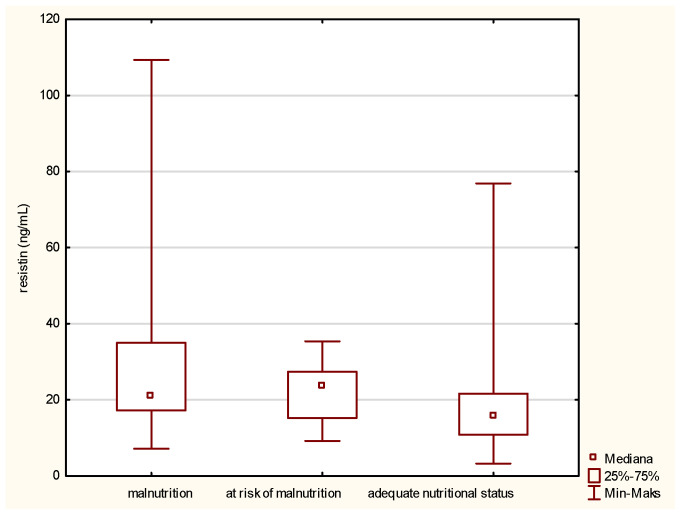
Concentration of resistin in the older patients having different nutritional status identified by the Mini Nutritional Assessment (MNA).

In the matter of resistin (Figure 2), its level is inversely related to the improvement of the patient nutritional status. A high concentration of resistin was noted in the fraction containing malnourished people. Along with the improvement of the nutritional status, designated using the MNA, the concentration of the tested adipokine was reduced.

### 3.2. Selected Oxidative Stress Parameters and Adipokines According to the GNRI

In the presented study, the GNRI was used as adjunct to the MNA questionnaire to assess the risk of nutrition-related complications. According to the GNRI, four subgroups of patients were distinguished as having high, moderate, low, and no risk of nutrition-related complications. Referring to the data presented in Table 4, statistically significant differences (marked in yellow) between the designated groups were identified for leptin and resistin, analogously to the MNA. However, additional parameters for which significant differences were determined include oxLDL and GPx.

**Figure 3:**
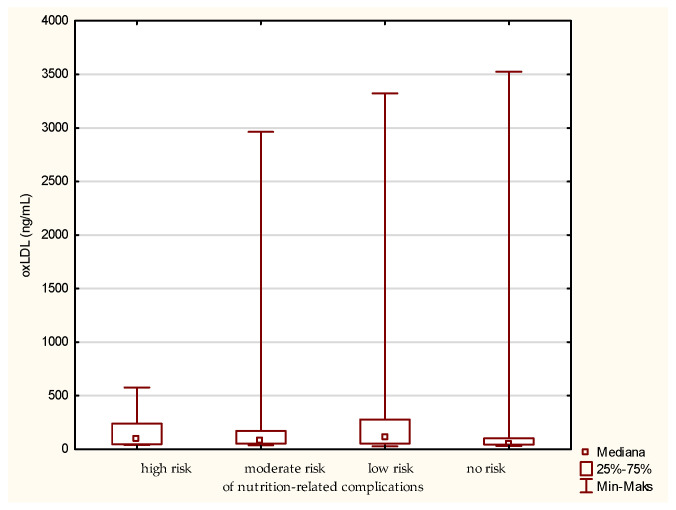
Concentration of oxidized low-density lipoprotein (oxLDL) in the older patients at different risk of nutrition-related complications defined by the Geriatric Nutritional Risk Index (GNRI).

For the level of oxLDL, the medians for the patients classified to the group of high, moderate, low, and no risk of nutrition-related complications are 96.87, 77.94, 105.62, and 55.42 ng/mL, respectively (Figure 3). Thus, the greatest difference is observed between the groups at low risk and no risk of nutrition-related complications, with the first one having the highest median. However, referring to the “no-risk” fraction, the highest individual oxLDL value was noted here. Surprisingly, in the group of patients at low risk of nutritional complications, the median is higher than in the patients at high and moderate risk. Moreover, in the group at high risk of nutritional complications, the smallest range of the assessed parameter was observed. Interestingly, grouping according to the MNA classification did not allow observing similar differentiation. Such results may indicate that the type of diet and other factors associated with the risk of atherosclerotic processes may be more significant for the level of oxLDL, and this parameter is not a good indicator of the risk of malnutrition. However, the explanation of such a distribution of the oxLDL variable requires a more detailed analysis on a larger group of older people.

**Figure 4:**
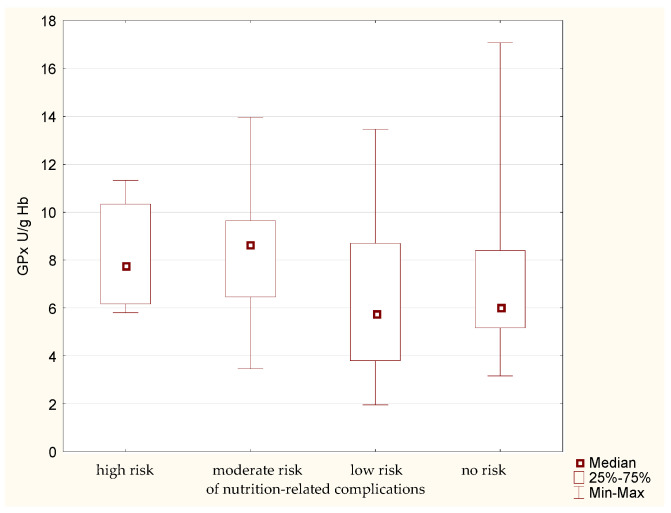
Activity of glutathione peroxidase (GPx) in the older patients having different risk of nutrition-related complications defined by the Geriatric Nutritional Risk Index (GNRI).

Analysis of GPx activity in the erythrocytes of the study participants allowed to determine certain regularities. The median erythrocytic activity of this enzyme (Figure 4) is higher in the groups of patients with high and moderate risk of nutrition-related complications. Lower enzymatic activity is noted in the other groups. The lack of confirmation of this phenomenon using the MNA implies the need to confirm the obtained results.

**Figure 5:**
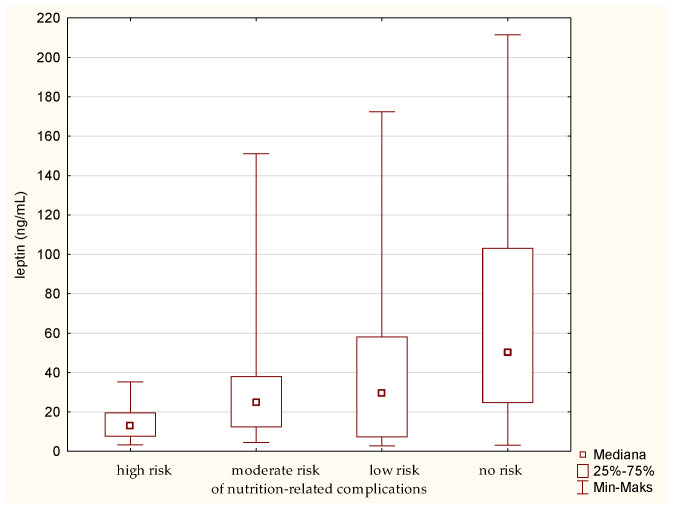
Concentration of leptin in the older patients at different risk of nutrition-related complications defined by the Geriatric Nutritional Risk Index (GNRI).

The analysis of leptin in relation to the GNRI showed a similar result as for the MNA. There was a statistically significant difference between the group of people at high risk and those without risk of nutrition-related complications. Leptin level is the highest in the fraction of people without a risk of nutrition-related complications. On the contrary, the lowest level of this parameter was determined in the group of senile patients with the highest risk of nutrition-related complications. In other words, the analysis of medians (Figure 5) indicates a gradual increase in the leptin concentration, along with a decrease in the risk of nutrition-related complications.

**Figure 6:**
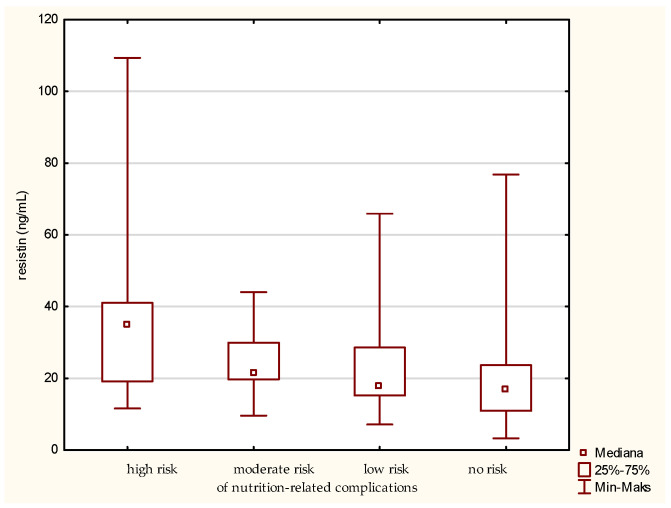
Concentration of resistin in the older patients at different risk of nutrition-related complications defined by the Geriatric Nutritional Risk Index (GNRI).

The highest level of resistin was determined in the older patients at high risk of nutrition-related complications (Figure 6). Significant differences were found between the groups designated by the GNRI as high risk and no risk of nutrition-related complications. The analysis showed the greatest difference between these groups. A gradual decrease in the concentration of resistin was also observed along with minimizing the risk of nutrition-related complications. The confirmation of this result was obtained by also using the MNA classification of the patients.

## 4. Discussion

The phenomenon of malnutrition in senile patients, although frequently underestimated, is one of the Giant Geriatric Syndromes, i.e., chronic and complex disorders that lead to functional disability. Malnutrition significantly reduces the quality of life of seniors [4]. Due to an aggregation of diseases and impairments, older adults tend to be more susceptible to nutritional deficiencies [30]. Surprisingly, over a half of patients at nutritional risk do not receive nutritional support or counselling, despite an active contact with healthcare professionals [31]. It generates the need to identify malnourished patients, especially during hospital stay, and implement appropriate nutritional management. For this purpose, it is necessary not only to routinely use screening tools for malnutrition, but also to search for new biomarkers.

In the present cross-sectional study of 137 patients admitted to the Geriatrics Clinic of the Antoni Jurasz University Hospital No. 1 in Bydgoszcz, Poland, as part of a comprehensive geriatric assessment, 34.06% were recognized as malnourished and 8.70% were at risk of malnutrition as classified by the MNA. The seriousness of the problem was also confirmed by complementary assessment of the risk of nutrition-related complications using the GNRI. This approach revealed that 36.5% of the study participants are at risk of complications (from low to high) related to the nutritional status. A similar number of malnourished patients was also identified in a single-center cross-sectional study by Son and Kavak [32], which included 102 individuals at the age of 65 years and older who were patients of the Family Health Center in the Cobanlar District in the Province of Afyon, Turkey. Out of 102 patients, whose nutritional status was evaluated with the use of the MNA scale, 38.2% were malnourished, 18.6% were at risk of malnutrition, and 43.1% displayed a normal nutritional status. Another cross-sectional study conducted in Saudi Arabia confirmed high occurrence of malnutrition among hospitalized senile patients [33]. As many as 248 hospitalized patients with an average age of 70.0 ± 7.7 years were recruited for that study and their nutritional status was assessed on the basis of the short form of Mini Nutritional Assessment (MNA-SF). In total, 76.6% of patients were at risk of malnutrition or undernourished. The accumulated data show the real scale of the problem in the daily practice of a geriatrician, indicating that practically every third patient manifests an abnormal nutritional status. Therefore, a possibility of malnutrition should be taken into account during a hospital stay from the perspective of diagnostics, pharmacotherapy, and making adequate nutritional recommendations. The incidence of malnutrition in hospitalized older adults depends on many factors and, in global assessment, the differences may be significant. Undoubtedly, malnutrition is a common problem among senile hospitalized patients, closely related to the length of hospital stay, as well as mortality in this group of patients.

In view of the above, finding a biomarker that adequately predicts nutritional status of a senile patient and is not influenced by inflammatory, fluid, or septic imbalance complications would facilitate the identification of hospitalized patients having abnormal nutritional status. In everyday practice, the MNA tool is the most frequently used to assess the nutritional status of patients. Additionally, the GNRI, used in the presented study as a complement to the MNA questionnaire, is one of the most commonly applied methods for identifying malnourished geriatric patients [34]. The quintessence of the conducted study was the analysis of selected parameters of oxidative stress and adipokines in relation to the nutritional status of recruited patients determined using the MNA, as well as to the risk of nutrition-related complications on the basis of GNRI (Table 3 and Table 4). Among ten parameters determined in the subjected patients, in the case of the MNA, differences at a statistically significant level were confirmed for two of them, namely leptin and resistin. Similarly, in the case of the GNRI, significant differences were also found for leptin and resistin, but, in addition, statistical differences were revealed for oxLDL and GPx.

Taking into account both classifications of the nutrition status, namely the MNA and the GNRI, leptin was found to vary significantly between the designed groups of senile patients. Leptin is a main peripheral messenger contributing to the regulation of food ingestion and energy balance. During starvation, the level of circulating leptin drops significantly, activating processes that increase food ingestion and decrease energy spending. The discovery of leptin as a hormone governing body mass and energy balance gave hope for a new approach to the treatment of obesity, involving its administration. Surprisingly, it has been shown that there are large levels of circulating leptin in the blood of people with obesity, which has been associated with the development of resistance to this hormone in people with excessive body mass [35]. In the presented study, the leptin concentration showed a downward trend along with the deterioration of the nutritional status, expressed both as the MNA score and the value of the GNRI. Significantly higher values were recorded in properly nourished patients compared to other groups, which is particularly illustrated by the GNRI (Figure 5). In other studies, a similar trend was observed and the possibility of using this marker in the assessment of the nutritional status of senile patients was indicated. These studies found a positive relationship between leptin levels and body fat mass in healthy individuals [36] or anthropometric indices [14,15], but no significant correlation with C-reactive protein [15]. Serum leptin may be a good prognostic factor of energy malnutrition, except for the cases of low creatinine clearance, diabetes, end-stage disease, or thyroid disease. As mentioned above, leptin levels decrease as nutritional status deteriorates. In comparison to the MNA tool, it may be an easier and more efficient method of recognizing energy malnutrition in older adults, because it does not depend on the subject’s memory and subjective judgment and allows avoiding systematic errors commonly made in anthropometric measurements. It can be especially useful for patients who cannot be weighed due to impaired motor skills or postural instability [15]. According to the presented study, leptin may be a promising biomarker of malnutrition of older patients. The trend is clearly visible, but a larger group of subjects and analysis according to the sex of involved patients are necessary to draw unambiguous conclusions. In fact, some authors suggested possible cut-off values of serum leptin depending on the sex of patients, i.e., approximately 4 ng/mL in men and 6.48 in women [14,15].

According to the presented study, resistin is also an adipokine related to the nutritional status of older patients, assessed with the use of both the MNA and GNRI scales. Resistin belongs to a family of cysteine-rich secreted proteins (the RELM family), newly discovered in 2001, related to the activation of inflammatory processes. It is also described as ADSF (adipose tissue-specific secretory factor) and FIZZ3 (a molecule found in inflammatory zones). The role of resistin was linked to the occurrence of obesity, insulin resistance, and diabetes in mice. However, no significant similarities in the structure and biological function of human and animal resistin have been shown in some studies [20,37]. Some authors indicated that plasma resistin levels are elevated in obese people, whereas other authors reported higher plasma resistin concentrations in athletes with high insulin sensitivity in comparison to obese people [20]. Resistin produced by human macrophages stimulates the production of TNF-α, IL-6, and interleukin 12 (IL-12) [19]. According to the latest reports, resistin is treated not only as a pro-inflammatory cytokine, but also as an atherogenic one. It is probably involved in inflammatory processes associated with obesity. However, its importance in physiological and pathophysiological conditions such as malnutrition is still uncertain [38]. Recent reports have suggested a pro-inflammatory impact of resistin on the development of dementia, especially in vascular dementia in geriatric patients [39]. However, abdominal obesity has not been found to have a significant effect on resistin levels in dementia patients. In the presented cross-sectional study, resistin levels were found to be inversely proportional to the patient’s nutritional status (Figure 2 and Figure 6). The literature on the subject mostly includes studies on resistin in patients with kidney disease [40]. For instance, in the study involving 80 senile patients, it was shown that the concentration of resistin increases with a decline in glomerular filtration rate (GFR), which may be related to malnutrition associated with the disease [41]. In non-diabetic patients with chronic kidney disease, a negative correlation between serum resistin concentration and albumin level as a marker of malnutrition was observed, which is consistent with the results of the presented study [41].

In the presented study, the significant differences in blood plasma oxLDL level were found between the groups of older patients classified by the GNRI. Oxidized low-density lipoprotein is regarded as an important biomarker of oxidative stress. It also plays a key role in the development of atherosclerosis. Increased plasma oxLDL concentration has been recognized as a prognostic indicator of mortality in the general population with congestive heart failure [42]. Dainy et al. [43] analyzed the relation of nutritional status, physical activity, and oxidative stress with cognitive function of 40 pre-elderly and 35 elderly subjects. The study revealed the prevalence of malnutrition in 60.0% and 80.0% of participants, respectively. However, only visual memory function was associated with oxLDL. Another study revealed a significant relationship between high oxLDL levels and cardiac systolic dysfunction in patients on continuous hemodiafiltration [44]. In the presented study, the highest level of oxLDL was determined in the older people with a high risk of nutrition-related complications, which may be associated with the highest level of oxidative stress and deficiency of antioxidant vitamins in the poor diet of this group of patients. In the patients with a low risk of nutrition-related complications, the level of oxLDL decreased and finally, in the patients with a moderate risk, the level of oxidative stress increased again. The lowest levels of oxLDL were recorded in the well-nourished subjects (Figure 3), which could be associated with an adequate supply of vitamins and other compounds with antioxidant activities. It is necessary to conduct a more thorough analysis on a larger group of people in order to confirm or exclude this phenomenon. Particularly, the results obtained in the case of the group with a low risk of nutrition-related complications are ambiguous. Obligatory research is also emphasized by the fact that variable grouping according to the MNA did not allow determining such a differentiation.

With regard to GPx, higher median enzyme activity was found in the senile patients at high and moderate risk of nutrition-related complications compared to low and no risk of such complications (Figure 4). In the patients at high risk of nutrition-related complications, the enzyme activity hardly decreased below 6 U/g Hb, which was observed in other groups. High GPx activity in patients at high risk of nutrition-related complications according to the GNRI might indicate the highest level of oxidative stress in this group of patients. Both current results and previous reports indicate that there is an inverse relation between oxidative stress and the nutritional status of older patients [9,45]. Moreover, severe oxidative stress is linked to a presence of metabolic syndrome in older individuals [46].

Malondialdehyde is a marker of the intensity of lipid peroxidation. Its increased level is observed in the adipose tissue of people with obesity [47]. In the conducted study, no statistically significant differences were observed between the groups determined by the MNA and the GRNI. Similar observations were made in a study of 69 older patients in a good health, living in the city [48]. No differences in the level of MDA were observed when examining the level of oxidative stress in relation to the control patients. However, an increase in the GPx activity was noted, as it was in the case of the presented study.

Superoxide dismutase and catalase are part of the antioxidant defense system. These antioxidant enzymes belong to the first line of defense against reactive oxygen species [49]. No statistically significant differences were determined according to the nutritional status for the abovementioned enzymes in the presented study. Similarly, in the study by Pinontoan et al. [50], no statistically significant differences were found for the erythrocyte SOD activity in non-frail and frail geriatric patients. The literature on the subject indicates an increase in oxidative stress markers (e.g., MDA) and a decrease in the activity of antioxidant enzymes (e.g., SOD and CAT) with the aging of the human body [9,51]. Although a relation between oxidative stress and the progression of the aging process is postulated, the influence of the nutritional status of senile patients on the activity of antioxidant defense enzymes has not yet been determined.

One of the parameters analyzed in the presented study is adiponectin, one of the most important adipokines secreted by adipose tissue. It possesses a strong anti-atherosclerotic and anti-inflammatory effect, lowers the level of glucose in the blood, and has a positive effect on the metabolism of carbohydrates and fatty acids [21,52]. Higher serum adiponectin level in older patients is accompanied, among other things, by a history of weight loss, low skeletal muscle mass, and poor physical functioning [53]. In the case of malnourished patients suffering from cancer, as shown by Bobin-Dubigeon et al. [54], increased adiponectin levels as well as decreased levels of leptin were observed. From the other point of view, Huang et al. [55] revealed that high serum adiponectin levels and low BMI were both linked to worsening depressive symptoms among older Japanese individuals. What is more, the combination of high adiponectin levels and low BMI was associated with worsening depressive symptoms. Although the adiponectin level was found the highest in the group at high risk of nutrition-related complications in the presented study, the lack of statistical significance does not allow drawing conclusions, and it is suggested to extend the study for a larger group of patients in the future.

Aging is accompanied by a considerable decrease in endogenous melatonin secretion, which exacerbates oxidative stress and induces other deleterious metabolic alterations [11]. Melatonin is a compound secreted by pinealocytes in a circadian rhythm. Impaired hormone secretion can lead to the disruption of the circadian rhythm of the secretion of adipokines related to satiety and hunger, such as leptin and ghrelin, leading to an excessive supply of energy. There are few results in the literature concerning the effect of melatonin supplementation on the normalization of body mass [11], but there is no information on the role of melatonin in malnutrition in seniors. In the presented study, no statistically significant relationship between the nutritional status of older hospitalized patients and the melatonin level was observed. Interestingly, Soysal et al. [56] found a close relationship between MNA scores and insomnia or insomnia severity in older adults. Patients suffering from insomnia had lower MNA scores than those without insomnia. Moreover, there were significant relationships between moderate/severe insomnia and the presence of malnutrition, the risk of malnutrition, and the MNA score.

## 5. Conclusions

The conducted study revealed relationships between the nutritional status of senile hospitalized patients and some of the analyzed parameters of oxidative stress and adipokines. Significant differences between the groups of patients identified on the basis of the MNA were confirmed for the serum levels of leptin and resistin. The additional division into groups regarding the GNRI not only confirmed this phenomenon, but also indicated two additional parameters, namely the serum level of oxidized low-density lipoprotein and the erythrocytic activity of glutathione peroxidase. In the studied population, a higher leptin concentration was related to an adequate nutritional status of the patient, whereas the resistin level was inversely related to the nutritional status. An analogous relationship was not observed in the case of adiponectin. Nevertheless, in the group of patients at high risk of nutrition-related complications, higher glutathione peroxidase activity and higher oxLDL concentration were found, indicating disturbed pro- and antioxidant processes in malnourished older people. Regrettably, the presented study does not allow indicating cut-off values for the identification of malnourished patients. However, the presented results encourage making further efforts in order to facilitate early identification of malnourished patients during hospitalization. Leptin, resistin, oxidized low-density lipoprotein, and glutathione peroxidase may be promising candidates for biomarkers of nutrition-related complications in older hospitalized patients, but this still needs to be confirmed on a larger group of subjects.

## Figures and Tables

**Table 1:** Nutrition status according to the Mini Nutritional Assessment questionnaire.

Nutritional Status	Malnutrition Indicator Score
malnutrition	less than 17 points
at risk of malnutrition	17 to 23.5 points
adequate nutritional status	24 to 30 points

**Table 2:** Risk of nutrition-related complications calculated with the use of the Geriatric Nutritional Risk Index (GNRI).

GNRI Score	Classification
<82	major risk
82–<92	moderate risk
92–≤98	low risk
>98	no risk

**Table 3:** Medians, results of the Kruskal–Wallis test and *p*-values of selected oxidative stress parameters and adipokine levels in the groups of older patients defined by the Mini Nutritional Assessment (MNA) scale.

Parameter	Malnutrition	Range	At Risk of Malnutrition	Range	Adequate Nutritional Status	Range	Total Average	Range	Kruskal–Wallis Test	*p* Value
MDApl ^1^ [nmol/mL]	0.5	0.5	0.5	0.3	0.5	0.5	0.5	0.5	H (2. N = 136) = 1.821581	0.4022
MDAer ^2^ [nmol/g Hb]	21.8	27.9	22.6	47.0	21.5	51.3	21.7	55.2	H (2. N = 137) = 0.7760118	0.6784
oxLDL ^3^ [ng/mL]	82.5	3295.2	50.7	391.3	55.8	3495.7	58.4	3498.8	H (2. N = 136) = 4.469296	0.1070
SOD ^4^ [U/gHb]	722.9	471.1	727.2	217.3	721.1	371.1	725.1	471.1	H (2. N = 137) = 0.0239938	0.9881
GPx ^5^ [U/gHb]	7.3	12.0	6.7	9.8	6.0	13.9	6.5	15.1	H (2. N = 137) = 1.293940	0.5236
CAT ^6^ [IU/gHb]	492,700.0	456,900.0	504,450.0	249,600.0	530,200.0	462,700.0	514,500.0	471000.0	H (2. N = 137) = 2.523735	0.2831
adiponectin [ng/mL]	14.6	34.0	14.1	29.5	12.8	32.4	13.5	35.6	H (2. N = 136) = 4.973683	0.0832
leptin [ng/mL]	18.5	169.7	29.7	198.6	52.5	208.4	35.8	208.8	H (2. N = 132) = 21.36475	0.0000
resistin [ng/mL]	21.2	102.2	23.7	26.1	15.9	73.6	18.5	106.1	H (2. N = 133) = 16.65061	0.0002
melatonin [pg/mL]	81.4	196.0	81.4	247.2	86.0	246.6	84.0	258.9	H (2. N = 136) = 0.3805290	0.8267

^1^ plasma malondialdehyde, ^2^ erythrocytic malondialdehyde; ^3^ oxidized low-density lipoprotein; ^4^ Zn/Cu-superoxide dismutase; ^5^ glutathione peroxidase; ^6^ catalase, yellow color—*p* < 0.05.

**Table 4:** Medians, results of the Kruskal–Wallis test and p-values of selected oxidative stress parameters and adipokine levels in the groups of older patients defined by the Geriatric Nutritional Risk Index (GNRI).

Parameter	High Risk	Range	Moderate Risk	Range	Low Risk	Range	No Risk	Range	Total Average	Range	Kruskal–Wallis Test	*p*
MDApl ^1^ [nmol/g Hb]	0.5	0.5	0.5	0.3	0.5	0.5	0.5	0.5	0.5	0.5	H (3. N = 136) = 4.754880	0.1907
MDAer ^2^ [nmol/g Hb]	22.2	21.6	20.2	18.0	21.8	48.7	21.6	51.3	21.7	55.2	H (3. N = 137) = 0.6137224	0.8933
oxLDL ^3^ [ng/mL]	96.9	536.8	77.9	2926.9	105.6	3295.2	55.4	3495.7	58.4	3498.8	H (3. N = 136) = 8.611890	0.0349
SOD ^4^ [U/gHb]	743.2	287.3	740.6	401.6	725.1	337.7	714.5	399.2	725.1	471.1	H (3. N = 137) = 1.634320	0.6516
GPx ^5^ [U/gHb]	7.8	5.5	8.7	10.5	5.7	11.5	6.0	13.9	6.5	15.1	H (3. N = 137) = 9.038221	0.0288
CAT ^6^ [IU/gHb]	511,850.0	319,200.0	525,000.0	335,600.0	484,900.0	456,900.0	524,000.0	462,700.0	514,500.0	471,000.0	H (3. N = 137) = 3.183901	0.3641
adiponectin [ng/mL]	20.6	34.0	14.6	27.0	10.5	24.4	13.4	35.0	13.5	35.6	H (3. N = 136) = 5.996740	0.1118
leptin [ng/mL]	13.0	31.9	24.8	146.6	29.5	169.7	50.2	208.4	35.8	208.8	H (3. N = 132) = 18.53068	0.0003
resistin [ng/mL]	35.0	97.7	21.5	34.5	17.5	58.8	16.6	73.6	18.5	106.1	H (3. N = 133) = 17.29766	0.0006
melatonin [pg/mL]	13.0	31.9	24.8	146.6	29.5	169.7	50.2	208.4	35.8	208.8	H (3. N = 132) = 18.53068	0.0003

^1^ plasma malondialdehyde, ^2^ erythrocytic malondialdehyde; ^3^ oxidized low-density lipoprotein; ^4^ Zn/Cu-superoxide dismutase; ^5^ glutathione peroxidase; ^6^ catalase; yellow color—*p* < 0.05.

## Data Availability

Data are available on request due to privacy/ethical restrictions.
